# Short-term effects by using additional methods in DOBOMED preparation phase for AIS double major patients – pilot study

**DOI:** 10.1186/1748-7161-7-S1-O58

**Published:** 2012-01-27

**Authors:** B Wnuk, J Durmala, J Dzierzega, K Dybula, S Dybula, K Wadolowski

**Affiliations:** 1 Medical University of Silesia, Poland, Katowice, Poland; 2 University Hospital - Silesia Medical Center, Katowice, Poland

## Aim

The assessment of physiotherapy various models to the clinical examination results – short-term observation during the stationary intensive rehabilitation.

## Materials and methods

Thirty-five girls with AIS double major (mean Cobb’s angle – Th=27o±7,5; L=24o±5,6) were divided for two randomized groups. In group A was applied only standard DoboMed [1].

In-group A-plus was applied triple method (DoboMed + OMT Kaltenborn-Evjenth + Dynamic Brace System –Meditrac). The derotation manual therapy techniques and Meditrac were used in DoboMed preparation phase. Meditrac was used only in the part of lumbar spine once a day during 30 minutes. The stationary intensive rehabilitation for both groups have been continued during 3 weeks.

Before and after observation have been analyzed: the respiratory system function (spirometry-VC, FEV1, PEF), the strength of respiratory muscles (maximal inspiration and expiratory pressures- MIP,MEP), the trunk morphology and function (kyphosis and range of spine motion by V-plurimeter; the trunk rotation angle-ATR by Bunnell’s scoliometer).

## Results

MIP and spine flexion values were increased significantly in both groups during therapy. In the group A-plus was observed more significant changes of parameters. Increasing of PEF, kyphosis, spine extension and lateral flexion values and decreasing of ATR were observed.

## Conclusions

In the short time were observed functionally and morphology improvement in-group of patients treated by DoboMed with OMT Kaltenborn-Evjenth and Dynamic Brace System –Meditrac. These additional methods have been used successfully in DoboMed preparation phase for AIS double major patients.
